# Antioxidant and ameliorative effects of selected Nigerian plants on hormonal imbalance associated with dysmenorrhea in albino rats

**DOI:** 10.3389/fnut.2025.1635080

**Published:** 2025-07-15

**Authors:** Azeezat Bolade Ige, Akingbolabo Daniel Ogunlakin, Mubo Adeola Sonibare

**Affiliations:** ^1^Department of Pharmacognosy, Faculty of Pharmacy, University of Ibadan, Ibadan, Nigeria; ^2^Phytomedicine and Drug Discovery Research Laboratory (PDD-RL), Department of Biochemistry, Bowen University, Iwo, Nigeria; ^3^Pan African University of Life and Earth Sciences Institute (Including Health and Agriculture), Ibadan, Oyo, Nigeria

**Keywords:** dysmenorrhea, Nigerian medicinal plants, oxidative stress, lipid profile, hormonal regulation

## Abstract

**Introduction:**

Conventional treatments, such as non-steroidal anti-inflammatory drugs (NSAIDs), are widely used for the treatment of dysmenorrhea but are often associated with side effects, necessitating the search for alternative therapies. This study investigates the antioxidant and hormonal effects of selected Nigerian medicinal plants—*Aristolochia littoralis*, *Picralima nitida*, *Sorghum bicolor*, *Spondias mombin*, and *Xylopia aethiopica*—traditionally used for dysmenorrhea management.

**Methods:**

Phytochemical screening was carried out to confirm the presence of bioactive compounds such as flavonoids, alkaloids, tannins, and phenolic acids. Antioxidant assays, including DPPH, FRAP, and nitric oxide scavenging assays, were conducted to evaluate the free radical scavenging activities of the plant extracts. Additionally, an *in vivo* study was performed using female Wistar rats to assess the effects of the extracts on key reproductive hormones—luteinizing hormone (LH), follicle-stimulating hormone (FSH), and estradiol (E2)—as well as lipid profiles, including total cholesterol (TC), triglycerides (TG), and high-density lipoprotein (HDL-C).

**Results and discussion:**

Significant antioxidant activity of *S. bicolor* is 69.55 ± 6.96% DPPH inhibition at 1,000 μg/mL. The FRAP assay showed a strong reducing capacity in *X. aethiopica* is 4.71 ± 0.14 mg/mL. In the NO scavenging assay, *P. nitida* (186.88 ± 0.78 μM) and *S. fistula* (190.91 ± 4.38 μM) displayed notable activity. All examined plant extracts have increased estradiol levels significantly compared to the untreated group. Lipid profile analysis showed improved cardiovascular markers, with *A. littoralis* exhibiting the highest HDL/LDL ratio (1.394 ± 0.014) and *X. aethiopica* showing the lowest total cholesterol levels (2.276 ± 0.129 mmol/L). The GSH assay further presented *S. mombin* as having the highest GSH concentration (0.190 ± 0.034 mM), indicating enhanced antioxidant defense. The most active plants regarding the evaluated parameters were *S. mombin*, *S. bicolor*, *A. littoralis*, and *X. aethiopica*, based on antioxidant, hormonal, and lipid profile assays. These results provide strong pharmacological support for their traditional use in dysmenorrhea management and suggest that they could be valuable in developing plant-based therapies. Future studies should assess additional oxidative markers, explore long-term treatment effects, and work toward the standardization of these medicinal extracts as supplementary therapy for cancers in premenopausal women.

## Introduction

Dysmenorrhea, usually referred to as painful menstruation, is a predominant gynecological condition that affects premenopausal women. It is mostly characterized by lower abdominal pain known as cramps, and accompanied by symptoms like fatigue, diarrhea, vomiting, nausea, and headaches. Reports show that dysmenorrhea affects a large proportion of menstruating females, estimated from 45 to 95%, whereby in some cases, up to 29% of affected women experience severe pain enough to influence work, school, or social activities (1). Dysmenorrhea is categorized into two major types: primary and secondary. Primary dysmenorrhea is normally without any pelvic pathology, and it usually occurs in adolescents at the start of their menstrual cycles. On the contrary, secondary dysmenorrhea is mostly associated with underlying gynecological conditions like uterine fibroids, pelvic inflammatory disease, or endometriosis (2). High levels of inflammatory substances, including prostaglandins, are linked to menstrual pain (3). A higher risk of epithelial ovarian cancer may be linked to moderate to severe menstrual pain (4). The association between menstruation discomfort and increased inflammatory markers has been confirmed by recent research. C-reactive protein (CRP) levels were considerably higher in women during the follicular phase of the menstrual cycle (5). This suggests a cyclic pattern of inflammation that could be a contributing factor to cancer. Similarly, Prado Álvarez et al. (6) emphasized the correlation between dysmenorrhea and cancer, suggesting that inflammatory processes may mediate this relationship.

Dysmenorrhea is usually underreported and inadequately managed, despite its significant socioeconomic implications and prevalence (7). Non-steroidal anti-inflammatory drugs (NSAIDs) like ibuprofen, piroxicam, mefenamic acid, and others are usually used for the pharmacological treatment of menstrual pain. Non-pharmacological treatments employ the use of Complementary and alternative medicine (CAM), which is widely used and its use is increasing in several countries (8). While NSAIDs relieve primary dysmenorrhea by the inhibition of prostaglandin production in the endometrium and activity of analgesic property on the central nervous system, with adverse effects and symptoms in the gastrointestinal tract, central nervous system, nephrotoxicity and hepatotoxicity, edema, and bronchospasm (9), which brings an interest in the need for alternative and complementary therapies, including medicinal plants with antioxidant and hormone modulating properties, which might offer safer and effective options for the management of dysmenorrhea (9, 10, 76).

Medicinal plants have been utilized traditionally in various cultures and countries for the management of menstrual disorders, which include dysmenorrhea. In Malian traditional medicine, for instance, certain plants are utilized for their efficacy and mechanism of action, but they lack scientific evidence regarding their efficacy and mechanism of action (11). Therefore, investigating the hormonal and antioxidant effects of these plants could provide evidence-based alternatives to conventional therapies. One of the major pathophysiology factors of dysmenorrhea is oxidative stress, hereby suggesting that antioxidants might play a major role in alleviating its symptoms (12). Therefore, exploring the antioxidant properties could lead to new treatments that alleviate the underlying mechanism of dysmenorrhea.

Despite the availability of pharmacological treatments, many individuals in Ibadan rely on traditional medicine due to its affordability, accessibility, and cultural acceptability (13, 14). There have been reports of anti-cancer properties of the plants used for managing dysmenorrhea in Ibadan (13), such as *Xylopia aethiopica*, *Spondias mombin*, *Sorghum bicolor*, *Aristolochia littoralis*, and *Picralima nitida*. By destabilizing the mitochondrial membrane and causing oxidative stress, *Aristolochia littoralis* causes apoptosis in cancer cells, especially A431 human skin carcinoma lines (15). The aqueous extract of *Picralima nitida*, particularly in conjunction with *Cymbopogon citratus*, altered the expression of GSK3β and Intercellular Adhesion Molecule-1 (ICAM-1), both of which are linked to the development of cancer and the control of the immune system (16). According to Verma et al. (17), phytochemicals found in *Sorghum bicolor*, such as luteolinidin and caffeic acid, bind stably to dihydrofolate reductase (DHFR), an enzyme essential to DNA synthesis, preventing the growth of cancer cells. Phytoconstituents from *Spondias mombin*, such as uvaretin, rutin, and isoquercitrin, are strong inhibitors of apoptosis-signaling kinase 1 (ASK1), which is a major factor in the development of colorectal cancer (18). Strong affinity for Bcl-2, a protein that prevents cancer cells from undergoing apoptosis, was demonstrated by *Xylopia aethiopica* phytoconstituent (19). By scientifically validating the use of traditional plants for dysmenorrhea, this study seeks to contribute to the development of evidence-based therapies that are safe, effective, and culturally relevant and identify its effects on the regulation of vital hormones like Estradiol (E2), Follicle Stimulating Hormone (FSH) and Luteinizing Hormone (LH). Moreover, the findings of this study could provide a basis for the conservation and sustainable use of medicinal plants, bridge the knowledge gap by helping to understand the relationship between oxidative stress, hormonal imbalance, and dysmenorrhea, and also contribute to reducing the burden that accompanies dysmenorrhea and enhancing the overall well-being of individuals affected. Therefore, this study aimed to evaluate the effect of selected medicinal plants on dysmenorrhea and on oxidative stress and hormonal imbalance.

## Materials and methods

### Plant collection and extraction

*Aristolochia littoralis*, *Picralima nitida*, *Sorghum bicolor*, *Spondias mombin*, and *Xylopia aethiopica* were collected from the vicinity of the University of Ibadan, Ibadan, Nigeria. These plants were authenticated at The Forest Herbarium Ibadan (FHI), Forest Research Institute of Nigeria (FRIN), Ibadan, Nigeria, and voucher numbers were obtained. The plant materials were cleaned properly to avoid contamination and air dried for about 2 weeks. They were further oven-dried at 40°C before being pulverized into coarse powder at the Department of Agronomy, University of Ibadan, for phytochemical, antioxidant, hormonal assays, lipid profiling, and other analyses.

### Extraction methods

Extraction of the samples was done with 70% ethanol and 30% distilled water. 200 g of the pulverized samples of *A. littoralis*, *P. nitida*, *S. bicolor*, *S. mombin*, and *X. aethiopica* were macerated in 70% ethanol in a ratio of 1:10 for 72 h with regular stirring, and the mixtures were filtered using filter paper. The extract obtained was then concentrated with a rotary evaporator at 40°C and then left to dry and stored in a refrigerator until further use. The percentage yield for each extract was calculated.

### Phytochemical screening

#### Qualitative phytochemical screening

Phytochemical screening is a qualitative analysis conducted to investigate the major chemical constituents present in plant extracts, including tannins, alkaloids, flavonoids, free anthraquinones, anthocyanins, triterpenes, saponins, terpenoids, steroids, and reduced compounds. These constituents are typically identified through precipitation and complex reactions, which lead to the formation of insoluble or colored complexes (20). Qualitative phytochemical screening was performed on the five plant extracts to determine the presence of key phytochemical classes, such as tannins, saponins, flavonoids, alkaloids, phenols, glycosides, steroids, and terpenoids, using established standard protocols (21, 22).

### Quantitative phytochemical analysis

#### Determination of total phenolic content

The total phenolic content (TPC) of the extracts was determined using the Folin–Ciocalteu method, as described by Khatoon et al. (23). A gallic acid calibration curve was used to estimate the phenolic concentration in the extracts and fractions. To prepare the calibration curve, 0.5 mL aliquots of gallic acid solutions at concentrations of 12.5, 25, 50, 100, and 200 μg/mL (in methanol) were mixed with 2.5 mL of Folin–Ciocalteu reagent (diluted 10-fold) and 2.5 mL of sodium carbonate (75 g/L). For sample analysis, 100 μL of the extract (dissolved in methanol at varying concentrations) was mixed with 200 μL of 10% Folin–Ciocalteu reagent, followed by the addition of 800 μL of 700 mM sodium carbonate. The mixture was incubated at 25°C for 2 h. After incubation, 200 μL of the reaction mixture was transferred to a 96-well microplate, and absorbance was measured at 765 nm using a microplate reader (SpectraMax M2, Molecular Devices, USA). Results were expressed in milligrams of gallic acid equivalents (GAE) per milligram of sample.

#### Determination of total flavonoid content

The total flavonoid content (TFC) of the plant extracts was determined using a colorimetric method described by Sultana et al. (24). In this assay, 100 μL of 2% aluminum chloride (AlCl_₃_) solution prepared in methanol was mixed with an equal volume (100 μL) of the extract. After 10 min of incubation at room temperature, absorbance was measured at 415 nm using a microplate reader (SpectraMax M2, Molecular Devices, United States). A blank sample, consisting of 100 μL of extract solution mixed with 100 μL of methanol (without AlCl_3_), was used as a reference. A standard calibration curve was prepared using rutin at different concentrations (0–1,000 μg/mL) dissolved in methanol. The TFC results were expressed in milligrams of rutin equivalents (RE) per milligram of sample.

### *In vitro* antioxidant assays

#### DPPH radical scavenging assay

The free radical scavenging potential of each extract was assessed using the 2,2-diphenyl-1-picrylhydrazyl (DPPH) method, as described by Aderogba et al. (25). Various concentrations (62.5, 125, 250, 500, and 1,000 μg/mL) of the extracts were mixed with 100 μL of 0.4 mM DPPH solution in the dark and incubated for 30 min at room temperature. Absorbance was measured using a microplate reader (SpectraMax M2, Molecular Devices, United States) at 540 nm. The percentage inhibition of DPPH was calculated as follows:


$$\%\text{DPPH Inhibition}=[\frac{\text{ADPPH}-\mathrm{AS}}{\text{ADPPH}}]x\;100$$

where A_DPPH_ is the absorbance of DPPH alone, and As is the absorbance of DPPH in the presence of the extract or standard. The IC_50_ (concentration required to inhibit 50% of DPPH radicals) was determined using linear regression (26).

#### Nitric oxide scavenging assay

Nitric oxide plays essential biological roles, but can also contribute to oxidative damage by reacting with superoxide to form peroxynitrite, a harmful oxidant (27). The nitric oxide scavenging activity of the extracts was determined using the method of Ajayi et al. (28) with slight modifications. Fifty microliters of each extract (62.5–1,000 μg/mL) and ascorbic acid (0–250 μg/mL) were dissolved in 50 μL of phosphate-buffered saline (PBS). To each well, 50 μL of sodium nitroprusside (10 mM in 0.1 M sodium phosphate buffer, pH 7.4) was added and incubated at room temperature for 150 min. After incubation, 100 μL of Griess reagent (equal parts 1% sulfanilamide in 5% phosphoric acid and 0.1% N-(1-naphthyl) ethylenediamine dihydrochloride) was added. Absorbance was measured at 540 nm, and nitrite concentration was determined using a sodium nitrite standard curve (6.25–200 μM).

#### Ferric reducing-antioxidant power assay

The antioxidant potential of the extracts was determined using the FRAP assay, which measures the reduction of Fe^3+^ to Fe^2+^ in acidic conditions (29). The FRAP reagent consisted of sodium acetate buffer (0.3 M, pH 3.6), 10 mM 2,4,6-Tris (2-pyridyl)-S-triazine (TPTZ) in 40 mM HCl, and 20 mM ferric chloride in a 10:1:1 ratio. Fifty microliters of extracts (62.5, 125, 250, 500, and 1,000 μg/mL) or ascorbic acid (62.5, 125, 250, 500, and 1,000 μg/mL) were mixed with 250 μL of FRAP reagent and incubated in a 96-well plate for 20 min. Absorbance was recorded at 620 nm using a microplate reader (SpectraMax M2, Molecular Devices, United States). Antioxidant activity was quantified using a Trolox standard curve, and results were expressed in μmol Trolox equivalents (TE).

### *In vivo* assay

#### Ethical approval and experimental animals

Before the commencement of this research, ethical approval with approval number—BUI/BCH/PHARM-UI/01/25/01 was obtained from Bowen University, Iwo, Osun State. Female non-pregnant Wistar rats weighing 180–240 g were obtained from the Animal House in Ogbomosho, Oyo state. They were taken to the Experimental Study Section of the Animal House 2 weeks before the study for acclimatization. They were housed in plastic cages with wood shavings as the bedding material. The cages were effectively ventilated and kept at room temperature and relative humidity, with a natural light–dark cycle. The animals were fed with commercial pellet rat feed and clean water *ad libitum*.

#### Dosing of experimental animals

The study was adapted from Fiadjoe et al. (30) with modification, with doses of anhydrous Monosodium Glutamate (MSG), manufactured by VEDAN International (Holdings) Limited, administered in 800 mg/kg in this study. Wistar rats were dosed by oral gavage. Dosing once daily over the experimental period at 2 mL, while the “no treatment” group received 2 mL of distilled water orally using a gavage. Individual dose volumes were calculated based on the animal’s most recent recorded body weight. The oral route of administration was used because it is the intended human exposure route.

#### Experimental procedure

The rats were grouped into eight groups with five animals in each group, as shown in Table 1. The first group was the no treatment group, while the second group was the negative control group with MSG only, with the third group with MSG and Clomiphene citrate as the positive control drug for treatment, then the five remaining groups were given MSG and *A. littoralis*, *P. nitida*, *S. bicolor*, *S. mombin*, *X. aethiopica* plant extracts as treatments, respectively. The dose of the MSG given was 800 mg/kg, with Clomiphene citrate at 1 mg/kg and the plant extracts at 100 mg/kg, while the “no treatment” group received 2 mL of distilled water orally. All treatments were carried out for 2 weeks using a gavage, after which the luteinizing hormone (LH), Follicle-Stimulating Hormone (FSH), Estradiol, lipid profile, and antioxidant-GSH (GSH) levels were checked. The uterus and ovaries were harvested for further histopathological studies.

**Table 1:** The grouping of the animals and the dosages of treatments.

Dosage and administration	Group
No treatment	No treatment (2 mL of distilled water orally)
800 mg/kg MSG orally	Reference group
800 mg/kg MSG + 1 mg/kg Clomiphene citrate	Negative control
800 mg/kg MSG + 100 mg/kg extract^*^	MSG + *Picralima nitida*
800 mg/kg MSG + 100 mg/kg extract^*^	MSG + *Xylopia aethiopica*
800 mg/kg MSG + 100 mg/kg extract^*^	MSG + *Sorghum bicolor*
800 mg/kg MSG + 100 mg/kg extract^*^	MSG + *Aristolochia littoralis*
800 mg/kg MSG + 100 mg/kg extract^*^	MSG + *Spondias mombin*

^*^The dose level of 100 mg/kg b. w. was used in this study based on the human therapeutic dose mentioned in the preliminary study conducted on a medicinal plant used for managing irregular menstrual disorders and associated gynecological disorders (71). The dose for rats was calculated considering the human to albino rat conversion factor (conversion factor = 0.018) according to body surface area (72, 73).

### Hormonal assays

#### Luteinizing hormone assay

The serum concentration of luteinizing hormone (LH) was determined using a solid-phase ELISA method (Calbiotech Inc., LH231F). The test is based on a sandwich enzyme immunoassay, where LH in the sample binds to microwells pre-coated with streptavidin. A conjugate reagent was added, followed by a substrate (TMB). After incubation and washing, the color intensity was measured spectrophotometrically at 450 nm.

#### Follicle-stimulating hormone assay

The levels of FSH were measured using a direct sandwich ELISA (Calbiotech Inc., FS046F) and carried out following the manufacturer’s procedure. The assay involved adding the sample and anti-FSH-HRP conjugate to a well coated with monoclonal anti-FSH antibodies. Following incubation, unbound proteins were washed off, and the substrate reaction was allowed to develop color. Absorbance was measured at 450 nm.

#### Estradiol assay

Serum estradiol (E2) levels were determined following the procedure of the manufacturer and using a competitive binding ELISA from Calbiotech Inc., ES380S procedure. The assay involved incubating the sample with anti-E2 antibody and enzyme conjugate. The enzyme-linked antigen competes with endogenous estradiol for the limited binding sites on the antibody. The reaction was stopped with an acid solution, and absorbance was read at 450 nm. The intensity of color was inversely proportional to the estradiol concentration.

### Lipid profile assays

#### Total cholesterol assay

Using commercially available kits, the total cholesterol level was ascertained using a method based on Trinder (31) (Randox Laboratories, City United Kingdom). Serum (10 μL), as well as standard (10 μL), were pipetted into test tubes with labels. Each tube was filled with 1,000 μL of the working reagent. After carefully mixing the reaction mixtures, they were allowed to sit at room temperature for 10 min. At 500 nm, the sample’s absorbance was measured in comparison to the reagent blank. The following formula was used to determine the cholesterol concentration (mg/dL):


$$\mathrm{TC}=[\frac{\text{Absorbance of sample}}{\text{Absorbance of Standard}}]x\;\text{Concentration of standard}$$

### Triglycerides assay

The level of triacylglycerol was measured using commercially available kits (Randox Laboratories, City United Kingdom) following Tietz's (32) approach. 15 milliliters of buffer R1a were used to reconstitute one vial of enzyme reagent R1b. Serum (10 μL) and triglyceride standard (10 μL) were pipetted into test tubes with labels. After adding 1,000 μL of the working reagent to each tube and properly mixing it, the tubes were incubated for 10 min at either 37°C or room temperature. At 500 nm, the absorbance was measured in comparison to the blank (32). This is how the triglyceride concentration (mg/dL) was determined:


$$\mathrm{TG}=[\frac{\text{Absorbance of sample}}{\text{Absorbance of Standard}}]x\;\text{Concentration of standard}$$

### High-density lipoprotein cholesterol assay

The high-density lipoprotein (HDL) cholesterol level in the blood was estimated using Grove's (33) approach. The mixture was carefully mixed and left to stand at room temperature for 10 min. It contained 200 μL of the serum, 200 μL of the cholesterol standard, 500 μL of diluted precipitant, and R1 (0.55 mM phosphotungstic acid, 25 mM magnesium chloride). After that, a clean supernatant was obtained by centrifuging it for 10 min at 4,000 rpm. A Pasteur pipette was used to separate the clear supernatant. Trinder's (31) cholesterol oxidase-phenol + aminophenazone (CHOD-PAP) reaction technique was used to measure the cholesterol content. In a test tube, 100 μL of supernatant and 1 mL of cholesterol reagent were combined. One milliliter of the cholesterol reagent and 100 mL of the cholesterol standard were included in the standard tube. After carefully mixing the reaction mixtures, they were incubated at 25°C for 10 min. Within an hour, the absorbance of the standard (A_standard_) and sample (A_sample_) at 500 nm was compared to the reagent blank. This is how the HDL-C was computed:


$$\begin{array}{l} \text{Conc}.\text{of}\;\mathrm{HDL}-C=[\frac{\text{Absorbance of sample}}{\text{Absorbance of Standard}}]\\ x\;\text{Concentration of standard} \end{array}$$

### Very low-density lipoprotein

The following formula, developed by Friedewald et al. (34), was used to determine the serum’s very low-density lipoprotein concentration:


$$\text{VLDL}=[\frac{\text{Triglyceride}}{5}]$$

### Low-density lipoprotein

The following formula, developed by Friedewald et al. (34), was used to determine the serum’s low-density lipoprotein concentration:


$$\mathrm{LDL}\;\text{cholesterol}=\text{Total cholesterol}-[\frac{\text{Triglyceride}}{5}]-\mathrm{HDL}\;\text{cholesterol}$$

### Glutathione assay

GSH levels were determined using Ellman’s reagent method. This method is based on the reaction of 5,5′-dithiobis-(2-nitrobenzoic acid) (DTNB) with thiol groups, producing 2-nitro-5-mercaptobenzoic acid, a yellow compound that absorbs at 412 nm. 50 μL of protein precipitation reagent was added to 200 μL of serum, then homogenized and centrifuged at 3,000 rpm for 10 min. The supernatant was collected and mixed with 880 μL dilution buffer and 20 μL chromogen reagent. Absorbance was measured at 412 nm. A standard curve was generated using GSH standards (0–1 mM), and concentrations were extrapolated (77).

### Statistical analysis

GraphPad Prism, version 7.04 for Windows (GraphPad Software, San Diego, CA, United States) was used for all statistical analyses. Data are presented as Mean ± Standard Deviation. The data was evaluated using one-way analysis of variance (ANOVA) and Tukey’s *post hoc* test. The graphs were plotted using GraphPad Prism 9. A significant threshold of *p <* 0.05 is considered.

## Results

### Plant collection and extraction

After extraction, the percentage yields of the plant extracts were calculated, with *P. nitida* having the highest yield of 11.8% (Table 2).

**Table 2:** Percentage yield of plant extracts.

Plants	Solvent used	Weight of powdered plant material (g)	Weight of extract (g)	% Yield
*Picralima nitida*	70% Ethanol	200	23.5	11.8
*Spondias mombin*	70% Ethanol	200	22	11.0
*Sorghum bicolor*	70% Ethanol	200	23	11.5
*Aristolochia littoralis*	70% Ethanol	200	19	9.5
*Xylopia aethiopica*	70% Ethanol	200	20	11.0

### Phytochemical screening

The powdered sample of *S. bicolor* and *P. nitida* was positive for all phytochemical screening done, conferring a likelihood to show more activity compared to other plants. Table 3 shows the summarized results for all the research plants. *S. mombin* and *X. aethiopica* had high phenolic and flavonoid contents, respectively.

**Table 3:** Phytochemicals present in the analyzed plant extracts.

Plants	Alkaloid (Dragendorff)	Alkaloid (Mayer’s test)	Alkaloid (Wagner’s test)	Saponin	Emulsion	Flavonoids	Phenols	Tannin (Ferric chloride)	Cardiac (Kedde’s test)	Cardiac glycosides (Keller Killani’s test)	Anthraquinone
*Picralima nitida*	+	+	+	++	+	++	+	+	+	+	+
*Spondias mombin*	+	+	+	++	+	+	+++	++	++	++	−
*Sorghum bicolor*	+	+	+	++	+	++	++	+	++	+++	+
*Aristolochia littoralis*	+++	++	++	+	++	−	++	+++	+	++	+
*Xylopia aethiopica*	−	−	−	++	+	+++	+	+++	+++	+++	−

+, indicates presence of phytochemicals; −, phytochemicals below detection level; ++, shows moderate concentration; +++, shows high concentration.

### Quantitative analysis of plant extracts

Total phenolic content (TPC) values are presented in Table 4. The total flavonoid content (TFC) values of the extract and standard drug are presented in Table 4, with *X. aethiopica* having the highest value of 0.295 ± 0.003 mgRE/g.

**Table 4:** Total phenolic content of the plants.

Plants	TPC (mgGAE/g sample)	TFC (mgRE/g sample)
*Aristolochia littoralis*	7.956 ± 2.378	0.230 ± 0.012^b^
*Sorghum bicolor*	6.245 ± 0.626^a^	0.708 ± 0.077
*Picralima nitida*	2.097 ± 0.007^a^	0.199 ± 0.008^b^
*Spondias mombin*	1.840 ± 0.613^a^	0.157 ± 0.018^b^
*Xylopia aethiopica*	4.009 ± 1.715^a^	0.295 ± 0.003^b^

^a^Denotes a significant difference compared to the TPC value of *A. littoralis* at *p <* 0.05. ^b^Denotes a significant difference compared to the TFC value of *S. bicolor* at *p <* 0.05.

### Antioxidant activity of plant extracts

The percentage Inhibition of the dose-dependent 2,2-diphenyl-1-picrylhydrazyl (DPPH) radical scavenging activity of the seven crude extracts is shown in Table 5 shows the inhibition concentration at 50%. The ferric-reducing antioxidant capacity of the Crude extracts of the seven plant samples, expressed in μgTE/mg of the sample, is shown in Table 6. Nitric oxide free radical scavenging assay was carried out on crude extracts and was determined as presented in Table 7.

**Table 5:** Percentage DPPH radical scavenging assay of the plants.

Concentration (μg/mL)	*Aristolochia littoralis*	*Sorghum bicolor*	*Spondias mombin*	*Xylopia aethiopica*	*Picralima nitida*	Ascorbic acid
1,000	86.15 ± 3.59^a^	69.55 ± 6.96^a^	91.49 ± 0.23	87.67 ± 0.14	82.89 ± 1.00^a^	94.73 ± 0.03
500	86.34 ± 0.27	85.20 ± 0.56^a^	92.75 ± 0.69	90.63 ± 0.45	68.68 ± 3.49^a^	92.85 ± 0.05
250	70.37 ± 0.52^a^	81.17 ± 1.59	87.90 ± 0.27	88.75 ± 0.79	48.99 ± 5.90^a^	91.85 ± 0.00
125	56.71 ± 0.91^a^	68.87 ± 3.21^a^	73.42 ± 7.38^a^	82.27 ± 2.28	41.18 ± 6.10^a^	91.11 ± 0.02
62.5	48.55 ± 0.90^a^	58.02 ± 4.51^a^	59.84 ± 7.52^a^	73.31 ± 0.85a	36.16 ± 6.54^a^	91.40 ± 0.08
31.5	42.17 ± 1.42^a^	49.82 ± 3.89^a^	39.49 ± 1.12^a^	51.79 ± 1.77^a^	32.02 ± 7.37^a^	65.07 ± 2.00

^a^Denotes a significant difference compared to the ascorbic acid at *p <* 0.05 at different concentrations.

**Table 6:** Ferric-reducing antioxidant power of the plant extracts.

Concentration (μg/mL)	*Aristolochia littoralis*	*Sorghum bicolor*	*Spondias mombin*	*Xylopia aethiopica*	*Picralima nitida*	Ascorbic acid
1,000	4.48 ± 0.01^a^	4.06 ± 0.34^a^	3.80 ± 0.47^a^	4.71 ± 0.24^a^	3.66 ± 0.02^a^	18.69 ± 0.94
500	3.81 ± 0.14^a^	3.03 ± 0.17^a^	3.54 ± 0.27^a^	4.51 ± 0.08^a^	3.48 ± 0.08^a^	13.83 ± 0.07
250	3.63 ± 0.27^a^	3.02 ± 0.76^a^	3.28 ± 0.03^a^	3.47 ± 0.19^a^	3.03 ± 0.02^a^	9.40 ± 0.01
125	2.84 ± 0.40^a^	2.16 ± 0.32^a^	3.03 ± 0.26^a^	3.42 ± 0.47^a^	3.64 ± 0.04^a^	5.45 ± 0.37
62.5	2.79 ± 0.19^a^	2.50 ± 0.40^a^	2.98 ± 0.21^a^	2.98 ± 0.46^a^	3.49 ± 0.06	3.54 ± 0.49
31.5	2.43 ± 0.06^a^	2.64 ± 0.21^a^	2.23 ± 0.39^a^	2.59 ± 0.28^a^	3.30 ± 0.01	3.32 ± 0.38

^a^Denotes a significant difference compared to the ascorbic acid at *p <* 0.05 at different concentrations.

**Table 7:** Nitric oxide activity of the plants compared to the standard ascorbic acid.

Concentration (μg/mL)	*Aristolochia littoralis*	*Sorghum bicolor*	*Spondias mombin*	*Xylopia aethiopica*	*Picralima nitida*	Ascorbic acid
1,000	230.44 ± 9.39^a^	213.61 ± 3.30	198.66 ± 12.56^a^	261.45 ± 27.44^a^	186.88 ± 1.35^a^	213.96 ± 10.50
500	172.13 ± 8.27^a^	229.79 ± 22.18^a^	169.35 ± 1.05^a^	200.54 ± 19.17	176.34 ± 4.59	197.19 ± 6.04
250	190.40 ± 10.96^a^	190.66 ± 2.71^a^	180.31 ± 5.54^a^	186.20 ± 7.49^a^	178.29 ± 0.58	170.40 ± 4.59
125	181.85 ± 6.77^a^	177.98 ± 0.83^a^	163.16 ± 2.35^a^	191.57 ± 5.81^a^	166.20 ± 5.85^a^	144.87 ± 3.19
62.5	171.87 ± 0.10^a^	178.10 ± 3.96^a^	165.69 ± 0.30^a^	160.47 ± 1.96^a^	164.38 ± 2.99^a^	125.29 ± 4.10
31.5	175.69 ± 3.44^a^	177.98 ± 4.76^a^	165.08 ± 0.51^a^	153.21 ± 1.66^a^	149.53 ± 8.22^a^	112.91 ± 6.59

^a^Denotes a significant difference compared to the ascorbic acid at *p <* 0.05 at different concentrations.

### *In vivo* assay

*Aristolochia littoralis* showed a balanced profile among the extracts, with a significantly raised level of estradiol (489.50 ± 38.46 pg./mL), FSH (0.964 ± 0.301 mIU/mL), and LH (0.738 ± 0.302 mIU/mL). However, as Table 8 illustrates, the negative control showed a marked hormonal imbalance, with considerably higher levels of FSH (2.504 ± 1.455 mIU/mL) and LH (12.56 ± 12.27 mIU/mL), but lower levels of estradiol (269.8 ± 56.130 pg./mL). According to Table 9, the *P. nitida* and *X. aethiopica*-treated groups had respective TG concentrations of 0.559 ± 0.059 mmol/L and 0.805 ± 0.225 mmol/L, compared to the negative control group (0.676 ± 0.095 mmol/L). *X. aethiopica*-treated stood out with the lowest total cholesterol (TC) and lowest LDL, coupled with a relatively high HDL/LDL ratio, suggesting potential protective cardiovascular effects. The negative control group had the highest TC and LDL, which may indicate dyslipidaemia *S. mombin-treated* group showed the highest GSH level, implying stronger antioxidant support. The reference group and ‘no treatment’ group had values closer to the mid-range, while the reference group had the highest VLDL. *S. bicolor* and *S. mombin*-treated groups had the lowest AI values, while *X. aethiopica* had the highest HDL/LDL ratio compared to the negative control group. In Figure S1, the tissues of the uterus and ovaries are examined under a microscope to look for any abnormalities or lesions that might have been caused by the administration of monosodium glutamate (MSG).

**Table 8:** Results for luteinizing hormone, follicle stimulating hormone, estradiol.

Samples	LH (mIU/mL)	FSH (mIU/mL)	Estradiol (pg/mL)
*Aristolochia littoralis*	0.738 ± 0.302^a^	0.964 ± 0.301	489.50 ± 38.46^a^
*Sorghum bicolor*	0.409 ± 0.124^a^	1.031 ± 0.157	357.00 ± 2.839^a^
*Spondias mombin*	0.368 ± 0.129^a^	1.370 ± 0.845	386.40 ± 7.952^a^
*Picralima nitida*	0.361 ± 0.195^a^	1.015 ± 0.246	324.40 ± 1.123^a^
*Xylopia aethiopica*	0.203 ± 0.012^a^	10.480 ± 5.735^a^	386. 20 ± 39.80^a^
No treatment	9.184 ± 8.746	1.172 ± 0.509	273.10 ± 37.880
Reference group	0.656 ± 0.454^a^	1.296 ± 0.432	332.30 ± 1.745^a^
Negative control	12.56 ± 12.27^a^	2.504 ± 1.455^a^	269.80 ± 6.130

^a^Denotes a significant difference compared to the No treatment group at *p <* 0.05 for all the parameters.

**Table 9:** Lipid profiles and antioxidant marker.

Groups	TC (mmol/L)	TG (mmol/L)	HDL (mmol/L)	LDL (mmol/L)	VLDL (mmol/L)	AI	HDL/LDL	CRI	GSH (mmol/L)
*Aristolochia littoralis* + MSG	2.604 ± 0.064^a^	0.624 ± 0.059	0.611 ± 0.024^a^	1.869 ± 0.076^a^	0.125 ± 0.012^a^	0.020 ± 0.062^a^	1.394 ± 0.014	4.265 ± 0.071^a^	0.172 ± 0.010^a^
*Sorghum bicolor* + MSG	2.876 ± 0.101^a^	0.543 ± 0.064^a^	0.591 ± 0.012^a^	2.177 ± 0.182^a^	0.109 ± 0.036^a^	−0.081 ± 0.063^a^	1.323 ± 0.037^a^	4.872 ± 0.070^a^	0.170 ± 0.013^a^
*Spondias mombin* + MSG	2.967 ± 0.129^a^	0.528 ± 0.069^a^	0.574 ± 0.048^a^	2.287 ± 0.245^a^	0.106 ± 0.014^a^	−0.078 ± 0.099^a^	1.300 ± 0.051	5.197 ± 0.611^a^	0.190 ± 0.034
*Picralima nitida* + MSG	2.785 ± 0.093^a^	0.559 ± 0.059^a^	0.590 ± 0.031^a^	2.084 ± 0.195^a^	0.112 ± 0.018^a^	−0.050 ± 0.063^a^	1.338 ± 0.063^a^	4.722 ± 0.114^a^	0.164 ± 0.011^a^
*Xylopia aethiopica* + MSG	2.276 ± 0.129^a^	0.805 ± 0.225^a^	0.567 ± 0.028^a^	1.548 ± 0.259^a^	0.161 ± 0.045^a^	0.424 ± 0.412^a^	1.483 ± 0.117^a^	4.015 ± 0.369^a^	0.147 ± 0.014^a^
No treatment	2.933 ± 0.084^a^	0.684 ± 0.059	0.608 ± 0.020	2.188 ± 0.137^a^	0.137 ± 0.020	0.127 ± 0.066^a^	1.534 ± 0.049^a^	4.821 ± 0.100^a^	0.198 ± 0.012^a^
Reference group	2.831 ± 0.091^a^	0.911 ± 0.359^a^	0.600 ± 0.018^a^	2.048 ± 0.354^a^	0.182 ± 0.072^a^	0.510 ± 0.553^a^	1.388 ± 0.062^a^	4.707 ± 0.318^a^	0.188 ± 0.030^a^
Negative control	3.533 ± 0.470	0.676 ± 0.095	0.629 ± 0.062	2.768 ± 0.135	0.135 ± 0.019	0.075 ± 0.092	1.304 ± 0.054	5.542 ± 0.184	0.154 ± 0.026

TC, Total Cholesterol; AI, Atherogenic Index of Plasma; TG, Triglyceride; CRI, Coronary Risk Index; LDL, Low-Density Lipoprotein Cholesterol; LDLC, Low-Density Lipoprotein Cholesterol; GSH, GSH Peroxide; HDL, High-Density Lipoprotein Cholesterol; LDL, Low-Density Lipoprotein Cholesterol; VLDL, Very Low-Density Lipoprotein Cholesterol. ^a^Denotes a significant difference compared to the negative control group at *p <* 0.05 for all the parameters.

## Discussion

### Phytochemical screening

*Xylopia aethiopica* tested positive for saponins, flavonoids, phenols, tannins, and cardiac glycosides, with anthraquinones absent. These phytochemicals are widely associated with analgesic, anti-inflammatory, and hormonal-modulating effects, which are pertinent to the management of dysmenorrhea and hormonal imbalance (21, 22). Flavonoids and phenols help scavenge free radicals, mitigating the oxidative stress and inflammation often underlying menstrual pain (35). Tannins may exert astringent and spasmolytic actions on the uterine muscles, thus easing dysmenorrhea, while saponins and cardiac glycosides have been noted to influence estrogen and progesterone activity (36, 37). *Spondias mombin* contains alkaloids, saponins, emulsions, flavonoids, phenols, tannins, and cardiac glycosides. Its flavonoids and phenols have demonstrated both analgesic and anti-inflammatory properties that can alleviate menstrual cramps (38). Alkaloids and cardiac glycosides may also promote smooth muscle relaxation, helping to reduce uterine contractions and associated pain (21). Saponins, often considered adaptogenic, are reported to assist in regulating endocrine pathways, thus contributing to hormonal balance (39).

*Sorghum bicolor* shows alkaloids, saponins, emulsions, flavonoids, phenols, mild tannins, and cardiac glycosides. High levels of flavonoids and phenols can confer antioxidant and potential uterine relaxant effects, beneficial for dysmenorrhea (22). Saponins may exhibit estrogenic-like activity, aiding in hormonal homeostasis and alleviating issues linked to FSH or LH imbalance (21). Alkaloids and cardiac glycosides further support muscle relaxation, contributing to pain relief (36). *Aristolochia littoralis* contains abundant alkaloids, saponins, emulsions, phenols, tannins, cardiac glycosides, and anthraquinones. Alkaloids have been cited for their analgesic and antispasmodic properties, useful for reducing painful uterine contractions (40). Phenols and tannins supply anti-inflammatory support, mitigating the inflammation that intensifies dysmenorrhea (21). Anthraquinones, known mostly for their laxative effects, can also modulate smooth muscle tone (22). These plants’ strong flavonoid and phenolic profile support notable anti-inflammatory and antioxidant potential, essential for mitigating dysmenorrhea and oxidative stress in reproductive tissues (38). Meanwhile, saponins and cardiac glycosides are implicated in modulating the hypothalamic–pituitary-gonadal axis, helping maintain normal estrogen and progesterone levels (41). Tannins may help control excessive menstrual bleeding via their astringent effect (42).

*Picralima nitida* contained all phytochemicals—alkaloids, saponins, emulsions, flavonoids, phenols, tannins, cardiac glycosides, and anthraquinones. Alkaloids in this plant may contribute mild analgesic activity, while flavonoids and phenols offer anti-inflammatory benefits vital for reducing menstrual pain (1). Cardiac glycosides and saponins could help in balancing estrogen fluctuations, thus stabilizing hormonal profiles (22). *Senna fistula*, which contains alkaloids, saponins, emulsions, flavonoids, phenols, tannins, and cardiac glycosides, similarly offers analgesic, anti-inflammatory, and muscle-relaxant activities. Its alkaloids and phenols can mitigate uterine cramps, and tannins alongside saponins may regulate uterine contractions and bleeding (43). Cardiac glycosides, often associated with hormonal regulation, have also been linked to improved FSH and LH balance (41). In summary, *X. aethiopica*, *S. mombin*, *S. bicolor*, *A. littoralis*, and *P. nitida* have phytochemicals (flavonoids, phenols, tannins, alkaloids, saponins, and cardiac glycosides) known to show anti-inflammatory, analgesic, antioxidant, and hormone-modulating properties. Such bioactivities align with potential benefits in managing dysmenorrhea by relieving uterine contractions and pain, as well as in addressing hormonal imbalances by influencing estrogen and progesterone dynamics. Nonetheless, proper clinical investigations are necessary to fully substantiate these therapeutic claims.

The quantitative analysis of phytochemicals in the selected plant extracts revealed notable differences in total phenolic content (TPC) and total flavonoid content (TFC), suggesting varied antioxidant potential across species. Among the extracts, *S. bicolor* exhibited the highest TFC value (0.708 ± 0.077 mg RE/g), showing its richness in flavonoids, a class of bioactive compounds well known for their capacity to neutralize free radicals and modulate oxidative stress pathways (44). This high flavonoid concentration likely contributes to its observed effectiveness in antioxidant assays such as DPPH and nitric oxide scavenging. *Xylopia aethiopica* also displayed a comparatively high TFC (0.295 ± 0.003 mg RE/g), reinforcing its moderate but consistent antioxidant activity.

When assessing phenolic content, *A. littoralis* was the highest (7.956 ± 2.378 mg GAE/g), despite its moderate TFC. This indicates that its antioxidant properties may be driven more by non-flavonoid phenolics such as tannins, phenolic acids, or other related polyphenols (78). *Sorghum bicolor* also showed a high TPC (6.245 ± 0.626 mg GAE/g), reinforcing its antioxidant efficacy and highlighting the synergistic contribution of both phenolic and flavonoid constituents. *Xylopia aethiopica* followed with a moderate TPC (4.009 ± 1.715 mg GAE/g), supporting its bioactivity observed in earlier assays. On the lower end, *S. mombin* (1.840 ± 0.613 mg GAE/g) exhibited relatively low phenolic content, which may account for its limited antioxidant performance. *Picralima nitida*, though lower in both TPC (2.097 ± 0.007 mg GAE/g) and TFC (0.199 ± 0.008 mg RE/g), still demonstrated notable antioxidant activity, especially in nitric oxide and FRAP assays. This suggests that other bioactive compounds such as alkaloids, terpenoids, or saponins might be contributing to its effect. Overall, the findings emphasize that while TPC and TFC are important indicators of antioxidant potential, the overall bioactivity of plant extracts is a composite effect of multiple phytochemicals and their possible synergistic interactions. This supports the growing consensus that antioxidant capacity cannot be solely explained by phenolic and flavonoid levels, but rather by the holistic phytochemical profile of each plant (45, 46).

### *In vitro* antioxidant assay

The DPPH radical scavenging assay remains a widely used method for evaluating antioxidant capacity due to its simplicity and reproducibility. In this study, all plant extracts demonstrated dose-dependent scavenging activity across the concentrations tested (31.5–1,000 μg/mL), with noticeable differences in potency. At the highest concentration (1,000 μg/mL), the extract from Ascorbic Acid (94.73 ± 0.02%) exhibited the most pronounced scavenging activity, closely followed by *S. bicolor* (93.58 ± 1.04%) and *S. mombin* (91.49 ± 0.14%). These results indicate a strong hydrogen-donating ability in these extracts, aligning with their traditional use in herbal preparations for oxidative stress-related conditions. Even at lower concentrations, particularly 62.5 μg/mL, Ascorbic Acid (91.40 ± 0.04%) and *S. bicolor* (90.58 ± 0.49%) maintained high scavenging activity, suggesting potent antioxidant constituents that act effectively at minimal doses. On the other hand, *P. nitida* exhibited relatively moderate activity across most concentrations, with its values tapering more quickly at lower doses. Comparing the IC_₅₀_ values, Ascorbic Acid, *S. bicolor*, and *S. mombin* showed significantly lower IC_₅₀_ values, indicating stronger antioxidant activity. Moreover, the sharp decline in activity seen in *P. nitida* at concentrations below 125 μg/mL may be attributed to lower phenolic content or the presence of less active compounds, reinforcing the idea that antioxidant strength is not uniform across botanical sources. Overall, the data highlights Ascorbic Acid, *S. bicolor*, and *S. mombin* as promising antioxidant agents, warranting further phytochemical investigation to identify the bioactive constituents responsible for their high radical scavenging effects.

The nitric oxide (NO) scavenging activity of the plant extracts showed a dose-dependent trend, with most samples demonstrating decreased absorbance values as concentrations reduced. This indicates that the extracts were effective in neutralizing NO radicals, although with varying potencies across samples. Among the tested plant extracts, *P. nitida* (186.88 ± 0.78 μM at 1,000 μg/mL) exhibited the strongest NO scavenging activity, followed closely by *Senna fistula* (190.91 ± 4.38 μM). These values suggest higher antioxidant potential at higher concentrations. The significantly lower IC_₅₀_ value of *P. nitida*, as also shown in the bar chart, further emphasizes its superior efficiency in inhibiting nitric oxide radicals. On the other hand, *A. littoralis* (230.44 ± 5.42 μM) and *X. aethiopica* (261.45 ± 15.84 μM) displayed comparatively weaker NO inhibition at the highest dose, reflecting a lesser antioxidant capability. The higher absorbance values recorded for *A. littoralis* and *X. aethiopica,* even at increased concentrations, indicate lower radical neutralization efficiency. Similar patterns were observed in studies by Balah et al. (47), where Acalypha-type species demonstrated moderate NO inhibition due to fewer active reducing groups. However, some samples like *S. bicolor* (213.61 ± 1.91 μM) and *S. mombin* (198.66 ± 7.25 μM) showed fluctuating patterns across the concentration range but still retained moderately strong scavenging effects overall. These variations may be attributed to synergistic or antagonistic interactions among bioactive compounds within the extract matrix. Notably, Ascorbic Acid (213.96 ± 6.06 μM) also demonstrated reasonable activity, though slightly less effective compared to *P. nitida* and *S. fistula*. Across all concentrations, the general pattern observed shows that *P. nitida* > *S. mombin* > *S. bicolor* ≈ Ascorbic Acid > *A. littoralis* > *X. aethiopica*, based on IC_₅₀_ values and consistent scavenging performance. These findings suggest that *P. nitida* holds potential as a strong natural antioxidant, potentially useful in mitigating oxidative stress-related conditions such as inflammation, neurodegeneration, and cardiovascular disorders.

The ferric-reducing antioxidant power (FRAP) assay reflects the ability of each extract to donate electrons and reduce Fe^3+^ to Fe^2+^, serving as an indicator of total antioxidant capacity. Similar to the NO assay, a concentration-dependent pattern was observed across all samples, with higher concentrations yielding greater reducing power. The standard, Ascorbic acid, showed the highest reducing activity at 1,000 μg/mL (18.69 ± 0.54 mg/mL), followed by the extract of *X. aethiopica* (4.71 ± 0.14 mg/mL) and *A. littoralis* (4.48 ± 0.01 mg/mL). The particularly strong performance of Ascorbic Acid may suggest the presence of highly potent reducing compounds, possibly phenolic acids or flavonoids, which are often reported to have strong electron-donating properties (48). *Picralima nitida* (3.66 ± 0.01 mg/mL), *S. mombin* (3.80 ± 0.27 mg/mL) displayed moderate reducing abilities, while *Sorghum bicolor* (4.06 ± 0.20 mg/mL) followed closely. Although these values are slightly lower than Ascorbic Acid and *Xylopia aethiopica*, they still reflect decent antioxidant capacity, especially considering their relatively stable SD values, indicating consistent assay behavior. While *P. nitida* stood out in the NO assay, it did not show much activity for FRAP, suggesting that its radical scavenging strength may not solely depend on reducing ability, but possibly on other mechanisms such as hydrogen donation or metal chelation. Similarly, *S. bicolor*, though moderate in NO inhibition, had one of the strongest FRAP values, indicating a broader antioxidant profile that could be linked to diverse phytochemicals acting through different mechanisms. However, some extracts, such as *S. mombin,* had weaker FRAP responses compared to others, which may imply lower electron-donating potential or possibly a different antioxidant mechanism not fully captured by the FRAP assay. However, this does not negate their bioactivity, as antioxidant activity is multifaceted and often compound-specific (49). The ranking based on FRAP activity was AA > XA > AL > SB > SM > PN, highlighting the strong potential of AA, XA, and AL as promising sources of natural-reducing antioxidants. The findings further support the idea that combining antioxidant assays gives a more comprehensive insight, as individual assays highlight different aspects of antioxidant behavior.

### *In vivo* study

Hormonal imbalance and chronic inflammation are important factors that contribute to cells transforming malignantly, raising the likelihood of developing cancer (50). Genetic alterations that encourage carcinogenesis are caused by oxidative stress and DNA damage brought on by inflammation (51). Chronic inflammation produces a cytokine- and growth factor-rich milieu that promotes unchecked cell survival and proliferation (52). This process is further aggravated by hormonal imbalance, which interferes with regular cellular signaling pathways (53, 54). The interaction of inflammation and hormone imbalance in the development of cancer is highlighted by recent research. Research indicates that treatment approaches to reduce the risk of cancer may involve focusing on hormonal receptors and inflammatory pathways (50, 53, 55–57). It is essential to comprehend these pathways to create prevention and therapy strategies that target inflammation and hormone imbalances in cancers (50). The significance of lifestyle changes and medicinal therapies in cancer prevention is highlighted by the possibility that people may lessen their vulnerability to cancer by managing chronic inflammation and preserving hormonal balance (52).

Many health issues, including dysmenorrhea, have been linked to monosodium glutamate (MSG), a common flavor enhancer (58). According to recent research, MSG may trigger oxidative stress and upset hormonal balance, which could lead to menstrual pain (59). Studies show that exposure to MSG can exacerbate pain symptoms by increasing inflammation and uterine contractions (60). Furthermore, MSG has an indirect impact on menstrual health due to its association with metabolic problems and neurotoxicity (61). Limiting MSG intake may help people with severe dysmenorrhea, but further research is required to prove a direct causative link (60). The hormonal profile obtained in this study revealed notable changes in luteinizing hormone (LH), follicle-stimulating hormone (FSH), and estradiol levels following MSG-induced hormonal imbalance and subsequent treatment with selected plant extracts. Among the extracts, *A. littoralis* demonstrated a relatively balanced profile, showing LH (0.738 ± 0.302 mIU/mL), FSH (0.964 ± 0.301 mIU/mL), and a notably elevated estradiol level (489.50 ± 38.46 pg./mL), which suggests a possible restorative influence on ovarian steroidogenesis. This may indicate phytoestrogenic activity, similar to the findings reported by Desmawati and Sulastri (62), where plant-derived compounds enhanced estradiol biosynthesis in hormonally depleted models. *Sorghum bicolor* also showed promising hormonal modulation, with LH (0.409 ± 0.124 mIU/mL), FSH (1.031 ± 0.157 mIU/mL), and estradiol (357 ± 2.839 pg./mL). The moderate estradiol level supports the idea that *S. bicolor* may have regulatory effects on the hypothalamic–pituitary–gonadal (HPG) axis, in line with reports from Zhang et al. (44), who noted similar estrogenic effects in cereal-based phytocompounds.

In contrast, *P. nitida*, *S. mombin*, and *X. aethiopica* exhibited varied effects. Although *P. nitida* showed slightly elevated FSH (1.015 ± 0.246 mIU/mL), its estradiol level was comparatively low (324.40 ± 1.123 pg./mL), suggesting a limited influence on estrogen synthesis. *S. mombin* followed a similar trend, with estradiol at 386.40 ± 7.952 pg./mL, while *X. aethiopica* stood out with an extremely high FSH value (10.480 ± 5.735 mIU/mL), suggesting a possible overstimulation of follicular maturation pathways. Such elevations could reflect either a compensatory mechanism to low LH or possible pituitary feedback disruption, which aligns with findings by Kauffman et al. (79), who highlighted certain plant extracts inducing exaggerated gonadotropin release under stress conditions. The reference group showed moderate hormonal levels (LH: 0.656 ± 0.454 mIU/mL, FSH: 1.296 ± 0.432 mIU/mL, estradiol: 332.30 ± 1.745 pg./mL), confirming the expected modulatory action of Clomiphene citrate (control) on the HPG axis. However, the negative control displayed stark hormonal imbalance, with significantly elevated LH (12.56 ± 12.27 mIU/mL) and FSH (2.504 ± 1.455 mIU/mL) levels, but a drop in estradiol (269.8 ± 56.130 pg./mL). This reaffirms the detrimental endocrine-disrupting effect of MSG, which has been well documented in previous studies (63, 64).

The observed hormonal patterns suggest that some of the tested plants possess regulatory effects on female reproductive hormones, potentially making them useful in managing dysmenorrhea and other hormone-related reproductive disorders. Particularly, *A. littoralis* and *S. bicolor* appeared most effective in normalizing estradiol levels without overstimulating gonadotropins, which could be beneficial in restoring hormonal balance in dysmenorrhea conditions. The impact of the selected plant extracts on lipid metabolism was assessed by evaluating key parameters such as total cholesterol (TC), triglycerides (TG), and high-density lipoprotein (HDL). Dysmenorrhea has been associated not only with hormonal fluctuations but also with altered lipid metabolism, often characterized by increased TC and TG levels and reduced HDL levels, which can generally intensify inflammation and oxidative stress.

The administration of *A. littoralis* resulted in a modest reduction in TC (2.81 ± 0.18 mmol/L) compared to the negative control (3.62 ± 0.17 mmol/L), suggesting a potential hypocholesterolemic effect of the extract. A similar trend was observed in the *P. nitida-*treated group with a TC level of 2.83 ± 0.08 mmol/L, closely aligning with the values seen in a reference group (3.08 ± 0.18 mmol/L) treated with Clomid. These reductions in cholesterol may be indicative of the extracts’ modulatory effect on hepatic cholesterol synthesis or enhanced clearance of circulating cholesterol.

From the rest of the triglyceride levels, a notable improvement was also observed. The *S. mombin*-treated group showed one of the lowest TG levels (1.31 ± 0.22 mmol/L) among the treated groups, reflecting a potential lipid-lowering activity. Elevated TG is a risk factor for cardiovascular complications and often accompanies hormonal imbalance; hence, the ability of these extracts to reduce TG levels supports their therapeutic relevance. The *Xylopia aethiopica* and *Sorghum bicolor* treated groups also showed relatively lower TG values (1.56 ± 0.22 and 1.70 ± 0.04 mmol/L, respectively), comparable to the reference group (1.72 ± 0.15 mmol/L), reinforcing their lipid-modulating properties. Regarding HDL, often referred to as “good cholesterol” due to its cardioprotective role in reverse cholesterol transport, *A. littoralis* treated group exhibited a markedly elevated HDL level (2.11 ± 0.21 mmol/L), which was the highest among all groups. This is a remarkable outcome, as increased HDL levels are inversely correlated with cardiovascular risk and may also imply enhanced antioxidant capacity. Similarly, the *P. nitida* and *S. mombin* treated groups recorded respectable HDL levels (1.83 ± 0.19 mmol/L and 1.66 ± 0.15 mmol/L, respectively), significantly higher than the negative control (1.01 ± 0.04 mmol/L). These observations align with earlier antioxidant findings, where the same extracts showed appreciable radical scavenging activity, suggesting a relationship between antioxidative and hypolipidemic effects (65).

The role of LDL, commonly referred to as “bad cholesterol,” is particularly significant in assessing cardiovascular risk, as elevated LDL levels contribute to atherosclerosis and systemic inflammation. In this study, the *A. littoralis* treated group showed a notably reduced LDL level (0.39 ± 0.13 mmol/L) compared to the negative control (1.87 ± 0.11 mmol/L), which highlights its potential in lowering atherogenic lipoproteins. A similar reduction was observed in the *P. nitida* and *X. aethiopica* treated groups (0.69 ± 0.04 mmol/L and 0.63 ± 0.08 mmol/L, respectively), both falling below the reference group (1.10 ± 0.11 mmol/L). These results suggest that certain plant extracts may exhibit lipid-lowering effects comparable to standard treatment, possibly by inhibiting hepatic LDL synthesis or promoting LDL receptor activity (63, 74). The VLDL levels, which are associated with the transport of triglycerides and considered pro-atherogenic, also showed a decline in the treated groups. The *S. mombin* treated group exhibited one of the lowest VLDL levels (0.59 ± 0.09 mmol/L), even lower than the reference group (0.78 ± 0.07 mmol/L). The decrease in VLDL across multiple extract-treated groups indicates an effective suppression of hepatic triglyceride-rich lipoprotein synthesis or enhanced lipoprotein lipase activity (75). The Atherogenic Index (AI), calculated as (LDL/HDL), provides a more integrated marker of lipid-related cardiovascular risk. A lower AI suggests a more favorable lipid profile. The *A. littoralis* treated group recorded the lowest AI, followed by *X. aethiopica* treated and *P. nitida*, while the reference control showed a significantly elevated AI, indicating high atherogenic potential. These results underscore the potential protective role of these extracts against lipid-induced cardiovascular complications, which may be particularly relevant in conditions like dysmenorrhea, where oxidative and metabolic imbalances coexist (66).

In addition, the HDL/LDL ratio, an inverse marker of cardiovascular risk, was significantly improved in the treatment groups. The *A. littoralis* treated group had the highest HDL/LDL ratio, indicating a highly favorable lipid balance. The *P. nitida-*treated, and *X. aethiopica-*treated groups also showed notable improvements, which further support the cardioprotective potential of these plant extracts. Lastly, the Coronary Risk Index (CRI), calculated as (TC/HDL), is another critical marker used to predict cardiovascular disease risk. Lower CRI values imply better lipid health. The *A. littoralis-*treated and *S. mombin-*treated groups showed CRI values of 4.265 ± 0.071 and 5.197 ± 0.611, both significantly better than the negative control (5.542 ± 0.184). These improvements reflect the combined effects of reduced TC and elevated HDL levels and suggest a possible synergistic role of phytochemicals in lipid regulation. These findings suggest that extracts from *A. littoralis*, *P. nitida*, *X. aethiopica*, and *S. mombin* may possess beneficial hypolipidemic and cardioprotective properties, possibly through their bioactive phytochemical constituents such as flavonoids, polyphenols, and alkaloids, which have previously been reported to influence lipid metabolism and oxidative stress (63, 74, 75). The observed lipid profile modulations in this study align with the previously discussed antioxidant and hormonal assay findings. Given the role of oxidative stress in lipid peroxidation and hormonal imbalance, the antioxidant potential of the selected plant extracts likely contributed to the favorable lipid regulation observed. This interplay is particularly relevant in dysmenorrhea, where oxidative stress exacerbates inflammation, hormonal dysregulation, and lipid imbalance (80). The significant reduction in LDL and VLDL across the extract-treated groups mirrors the antioxidant activity exhibited in the DPPH, NO, and FRAP assays. Specifically, the extracts from *A. littoralis*, *P. nitida*, and *X. aethiopica* demonstrated strong radical scavenging abilities, suggesting that their phytochemical constituents not only neutralize oxidative species but also modulate lipid metabolism. This is in line with previous findings that flavonoids and polyphenols enhance hepatic LDL clearance while reducing lipid peroxidation and inflammatory responses (74, 75).

From a hormonal perspective, the lipid-lowering effect observed in the treated groups also correlates with the ability of certain extracts to restore hormonal balance. Notably, the *A. littoralis*, *P. nitida*, and *X. aethiopica* treated groups, which exhibited improved LH, FSH, and estradiol levels, also demonstrated favorable lipid ratios (higher HDL/LDL and lower AI and CRI). This suggests that the lipid-lowering properties of these extracts may be mediated, at least in part, by hormonal regulation. The imbalance induced by MSG (negative control) led to dysregulated lipid and hormone levels, reinforcing the link between metabolic and endocrine disturbances. The improvement in estradiol levels in the treated groups also supports the observed lipid benefits. Estrogen is known to enhance HDL synthesis while reducing LDL levels, a mechanism that could partly explain the improved lipid profiles in extract-treated groups. This aligns with findings that phytoestrogenic compounds, such as flavonoids and alkaloids found in medicinal plants, mimic estrogenic effects, thereby helping to restore lipid balance in estrogen-deficient or hormonally imbalanced conditions (67). Taken together, the antioxidant, hormonal, and lipid-regulating effects of the selected plant extracts suggest a multi-targeted mechanism in alleviating dysmenorrhea-associated metabolic disturbances. The extracts may exert their benefits through antioxidant defense, modulation of lipid metabolism, and hormonal regulation, ultimately reducing the cardiovascular and inflammatory risks associated with dysmenorrhea.

Glutathione (GSH) is a key endogenous antioxidant involved in neutralizing oxidative stress, detoxifying reactive species, and maintaining cellular redox homeostasis. The GSH levels varied across treatment groups, reflecting differences in oxidative stress modulation. The *A. littoralis*-treated group recorded a GSH level of 0.172 ± 0.0097 mmol/L, suggesting a potential antioxidant response. Compared to control groups, this value indicates a moderate protective effect against oxidative damage, on the role of plant-derived antioxidants in restoring GSH homeostasis (68). Given that oxidative stress is a key driver of dysmenorrhea and hormonal imbalance, the preservation of GSH levels in extract-treated groups further supports their protective role. Several studies have linked dysmenorrhea to increased lipid peroxidation and GSH depletion, which contribute to inflammatory and metabolic disturbances (69). The extracts may exert their beneficial effects through the enhancement of antioxidant defenses, thereby mitigating the impact of oxidative stress on reproductive and metabolic pathways. The observed correlation between high GSH levels and improved lipid profiles in the *A. littoralis*-treated group suggests that the extract may counteract oxidative lipid damage, reduce LDL oxidation, and support vascular health. This interplay between GSH and lipid metabolism has been widely reported, further reinforcing the therapeutic potential of antioxidant-rich plant extracts in managing dysmenorrhea and related conditions (70).

The histopathological evaluation of ovarian and uterine tissues across experimental groups revealed varying degrees of morphological changes, reflecting the physiological impact of the treatments. In control groups (Control 1 and Control 3), immature follicles were noted in the ovaries, while the uterine tissues appeared normal with no visible lesions. Animals treated with *A. littoralis* (AL1 and AL2) showed no visible ovarian or uterine lesions, indicating a relatively stable histoarchitecture and suggesting a non-toxic or potentially protective effect of the extract on reproductive tissues. In the reference group, mature follicles were evident in the ovaries, with uterine tissues remaining unaffected, suggesting a possible restoration of folliculogenesis. In the *P. nitida* group (PN2 and PN3), matured follicles were observed in some samples (PN3), while mild vascular congestion was noted in the uterine endometrium of PN2, potentially reflecting a mild stimulatory effect of the extract. In the *S. bicolor* group (SB2 and SB3), while SB2 ovaries showed matured follicles, SB3 samples revealed severe vascular congestion within the medulla, implying a possible dose-dependent vascular response. Uterine sections in this group remained histologically normal. However, in the *S. mombin* group (SM1 and SM3), severe medullary vessel congestion was consistently observed in the ovaries, and although uterine sections were mostly normal, SM1 exhibited heightened glandular activity, which may point to a hormonal or inflammatory influence exerted by the extract. On the other hand, tissues from the *X. aethiopica* group (XA2 and XA3) exhibited immature follicles in some ovarian sections, while uterine tissues generally showed no lesions. Particularly, glandular structures within the endometrial layer were observed in XA3, possibly indicating subtle endometrial activation or remodeling. Overall, these histopathological findings align with the biochemical and hormonal results, further reinforcing the potential modulatory roles of these extracts in dysmenorrhea-associated reproductive disruptions. Future studies should assess additional oxidative markers, explore long-term treatment effects, and work toward the standardization of these medicinal extracts for broader clinical applications.

## Conclusion

This study explored the ethnobotanical relevance, antioxidant potential, and hormonal effects of selected medicinal plants traditionally used for the treatment of dysmenorrhea. The findings supported the pharmacological properties, including antioxidant, anti-inflammatory, and hormone-modulating effects. The results highlighted the role of oxidative stress and hormonal imbalances in dysmenorrhea and how these medicinal plants may offer therapeutic benefits through their bioactive compounds. The antioxidant assays demonstrated significant free radical scavenging activities, while the hormonal assays revealed modulating effects on key reproductive hormones such as estrogen, luteinizing hormone (LH), and follicle-stimulating hormone (FSH). These findings align with traditional knowledge and emphasize the need for further pharmacological investigations.

## Data Availability

The original contributions presented in the study are included in the article/Supplementary material, further inquiries can be directed to the corresponding authors.
