# Supplementary material for: Antioxidant and ameliorative effects of selected Nigerian plants on hormonal imbalance associated with dysmenorrhea in albino rats
Source: Front Nutr. 2025 Jul 15;12:1635080. doi: 10.3389/fnut.2025.1635080 (PMC12303999; doi:10.3389/fnut.2025.1635080)
Supplement: Supplementary file 1 [file Data_Sheet_1.docx]

**Antioxidant and ameliorative Effects of selected Nigerian Plants on hormonal Imbalance associated with Dysmenorrhea in Albino Rats**

**Azeezat Bolade Ige^1^, Akingbolabo Daniel Ogunlakin^2*^, Mubo Adeola Sonibare^1,3*^**

^1^Department of Pharmacognosy, Faculty of Pharmacy, University of Ibadan, Ibadan, Nigeria.

^2^Phytomedicine and Drug Discovery Research Laboratory (PDD-RL), Department of Biochemistry, Bowen University, Iwo, 232101, Nigeria.

^3^Directorate, Pan African University of Life and Earth Sciences Institute (including Health and Agriculture), Ibadan, Oyo state, Nigeria.

*Corresponding authors: [gbolaogunlakin@gmail.com](mailto:gbolaogunlakin@gmail.com); [sonibaredeola@gmail.con](mailto:sonibaredeola@gmail.con)

**Supplementary data**

**
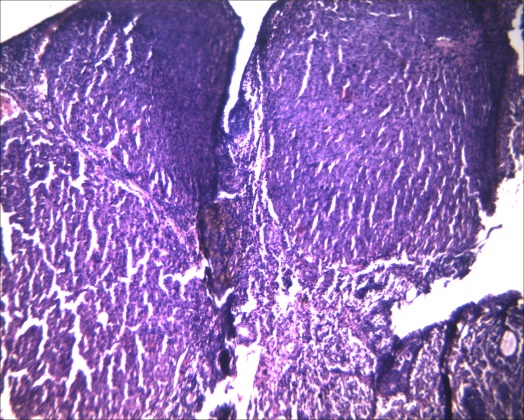
**

**Clomid + MSG 1 Ovary:** Immature follicles seen (arrows)


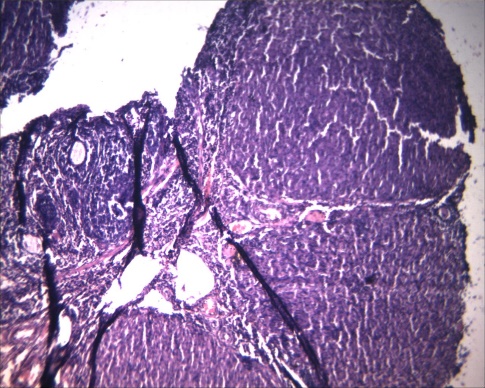


**Clomid + MSG 3 Ovary:** Immature follicles seen (arrows)


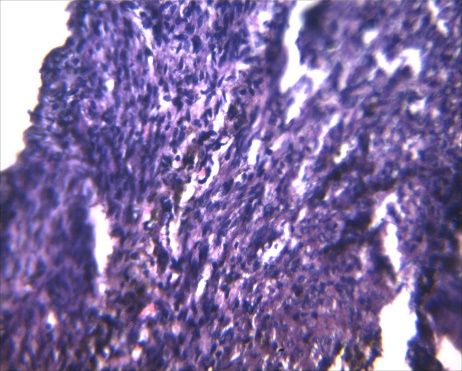


**Clomid + MSG 1 Uterus:** No visible lesions seen.


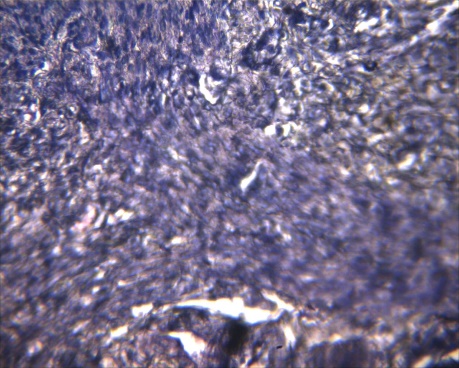


**Clomid + MSG 3 Uterus:** No visible lesions seen.


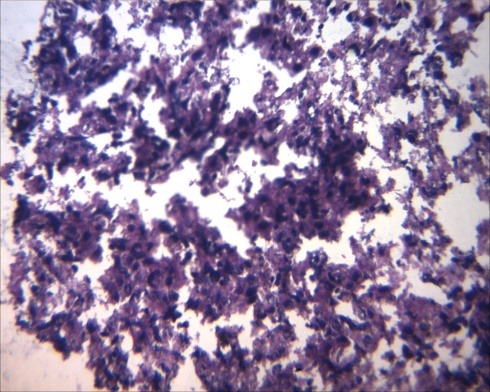


***Aristolochia littoralis* 1 Ovary:** No visible lesions seen.


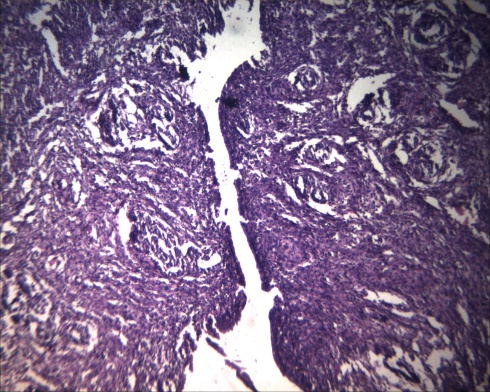


***Aristolochia littoralis* 2 Ovary:** No visible lesions seen.


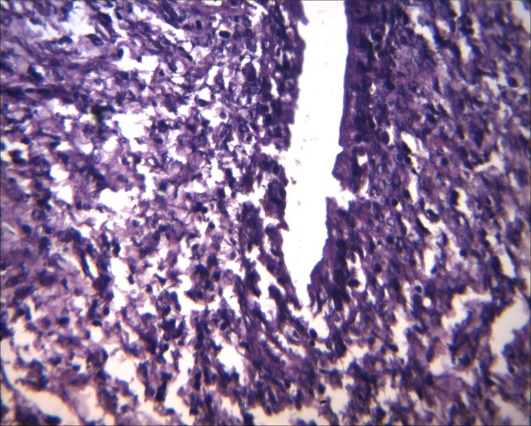


***Aristolochia littoralis* 1 Uterus:** No visible lesions seen.


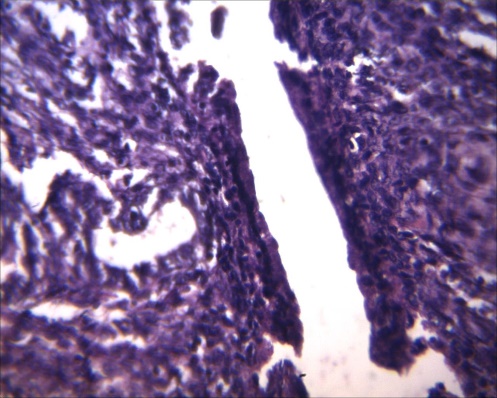


***Aristolochia littoralis* 2 Uterus:** No visible lesions seen.


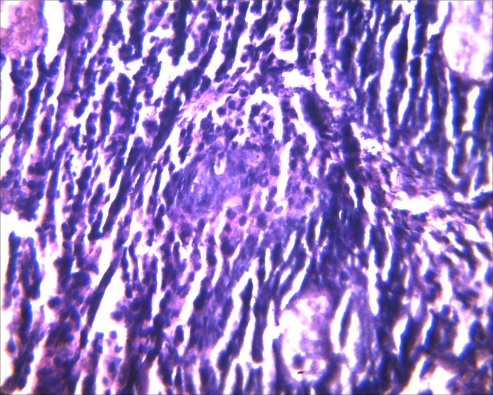


**Control 1 Ovary:** No visible lesions seen.


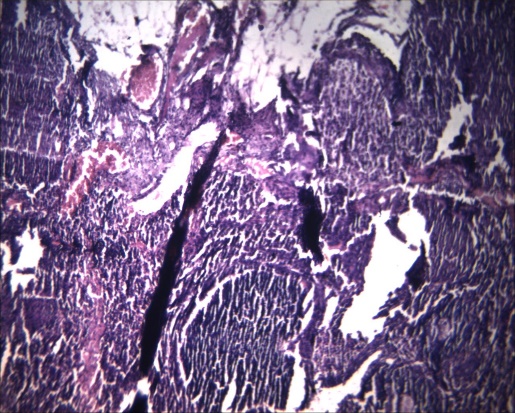


**Control 3 Ovary:** No visible lesions seen.


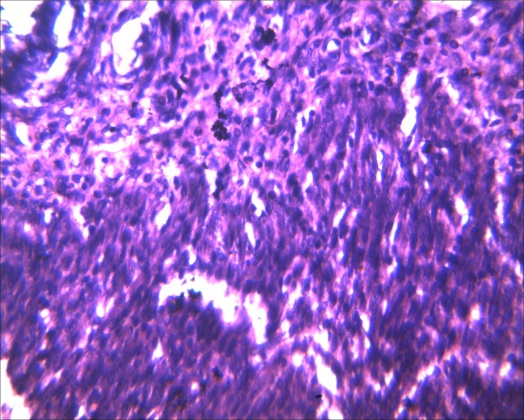


**Control 1 Uterus:** No visible lesions seen.


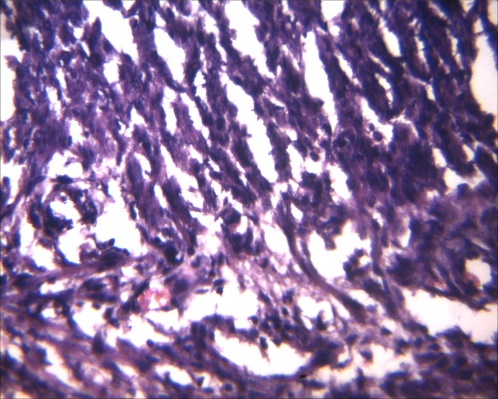


**Control 3 Uterus:** No visible lesions seen.


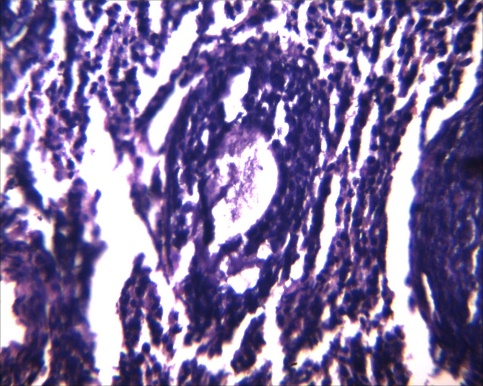


**MSG only Ovary:** No visible lesions seen. Matured follicle seen (arrows).


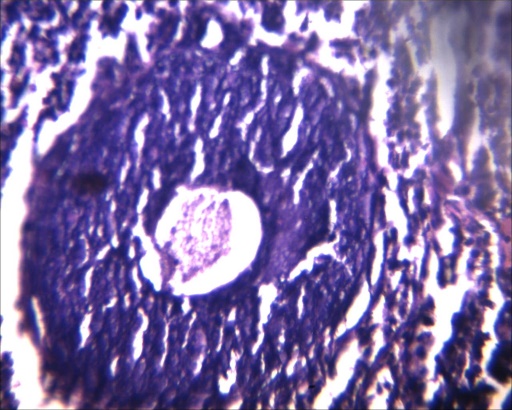


**MSG only Ovary:** No visible lesions seen. Matured follicle seen (arrows).


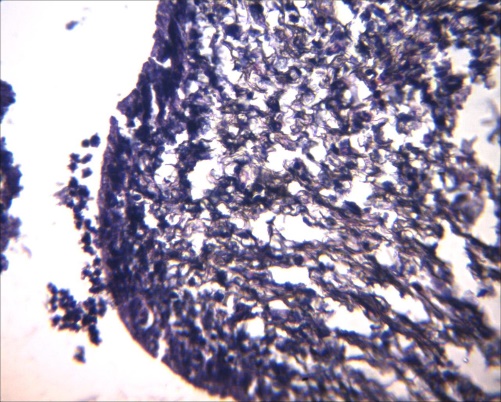


**MSG only 1 Uterus:** No visible lesions seen.


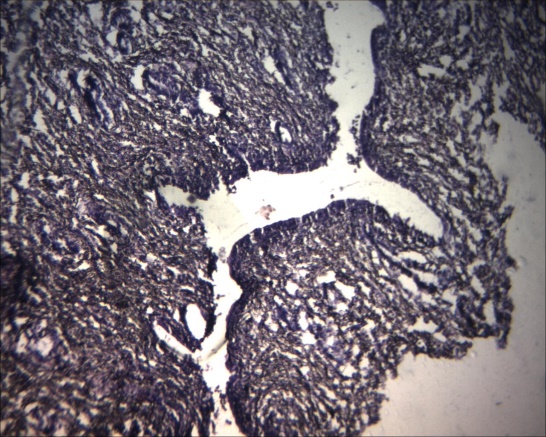


**MSG only 3 Uterus:** No visible lesions seen.


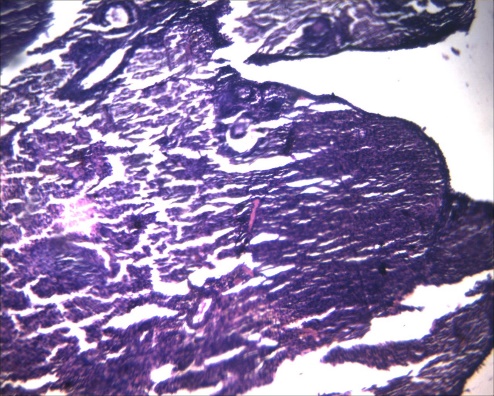


***Picralima nitida* 2 Ovary:** No visible lesions seen.


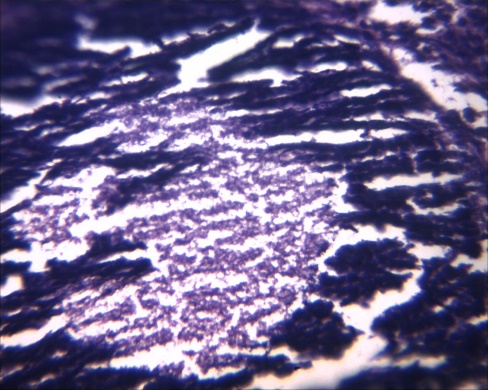


***Picralima nitida* 3 Ovary:** No visible lesions seen. Matured follicles seen (arrows)


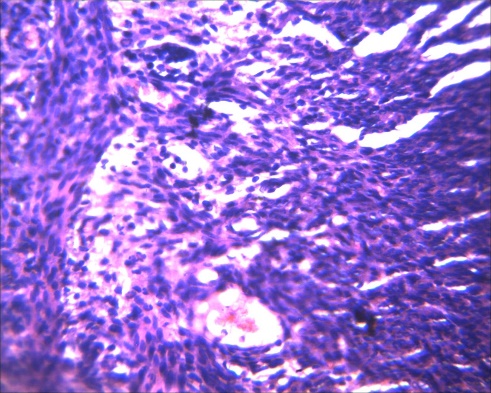


***Picralima nitida* 2 Uterus:** Few endometrial vessels appear congested (arrows)


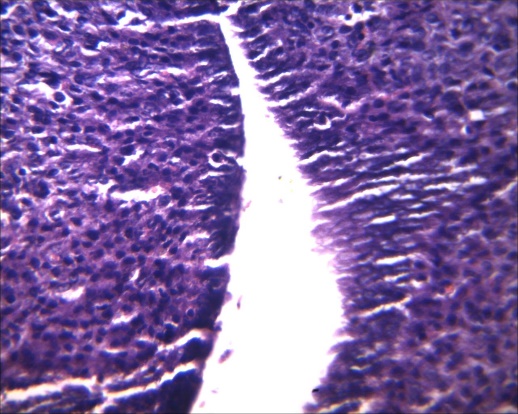


***Picralima nitida* 3 Uterus:** No visible lesions seen.


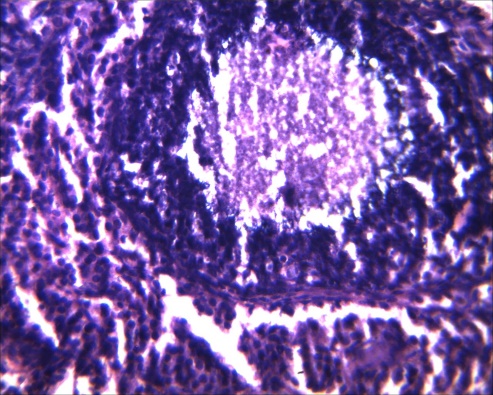


***Sorghum bicolor* 2 Ovary:** No visible lesions seen. Matured follicle observed (arrows)


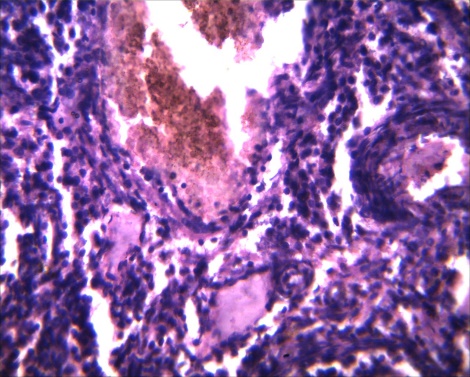


***Sorghum bicolor* 3 Ovary:** The medullary vessels are severely congested (arrows)


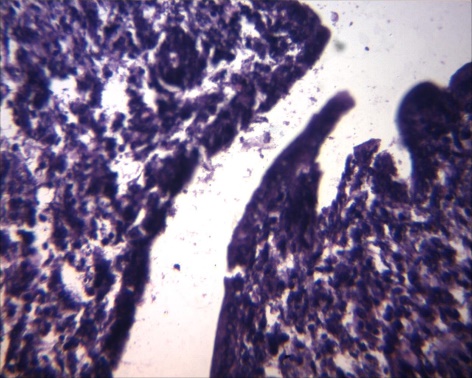


***Sorghum bicolor* 2 Uterus:** No visible lesions seen.


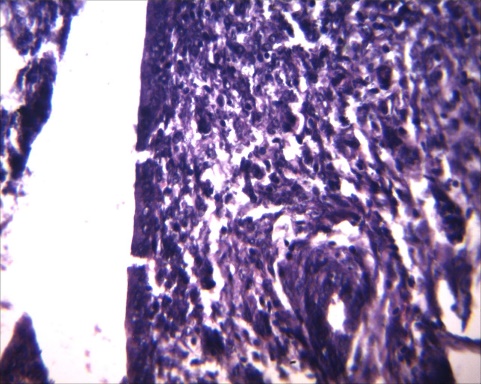


***Sorghum bicolor* 3 Uterus:** No visible lesions seen.


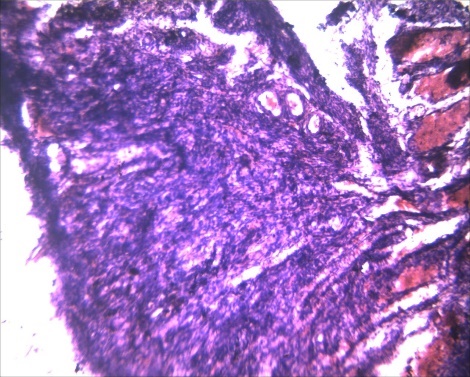


***Spondias monbin* 1 Ovary:** The medullary vessels are severely congested (arrows)


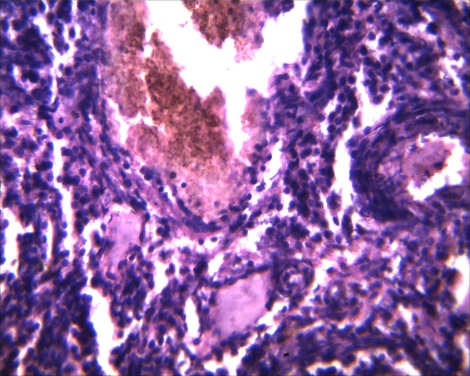


***Spondias monbin* 3 Ovary:** The medullary vessels are severely congested (arrows)


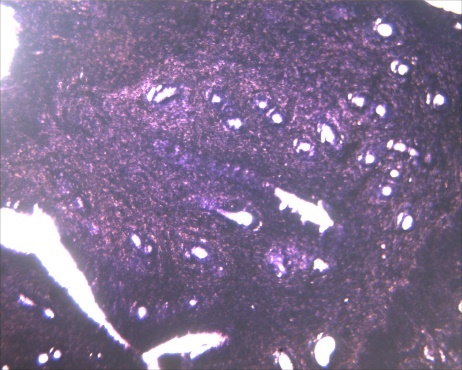


***Spondias monbin* 1 Uterus:** No visible lesions seen. There is a considerable glandular activity (arrows)


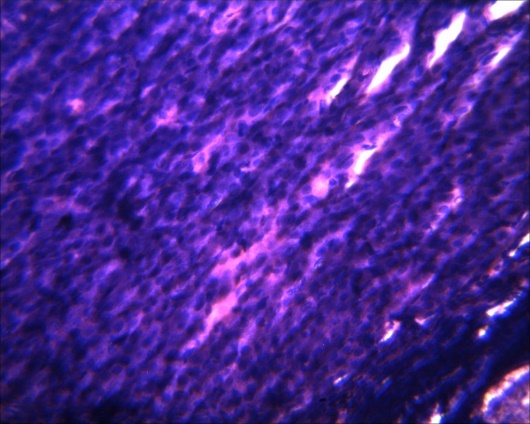


***Spondias monbin* 3 Uterus:** No visible lesions seen.


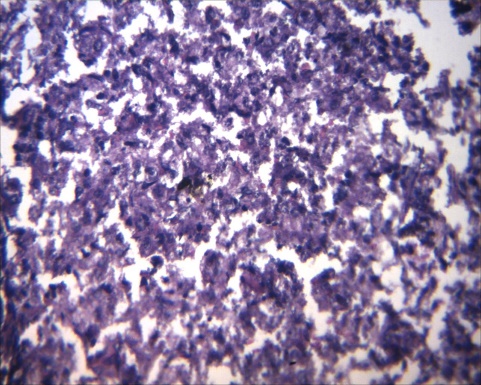


***Xylopia aethiopica* 2 Ovary:** No visible lesions seen.


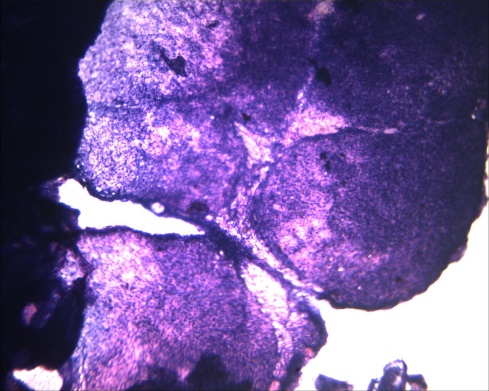


***Xylopia aethiopica* 3 Ovary:** No visible lesions seen. Immature follicles observed (arrows)


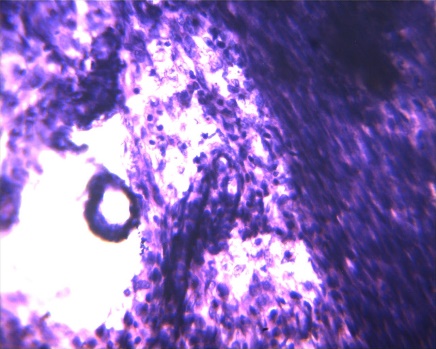


***Xylopia aethiopica* 1 Uterus:** No visible lesions seen.


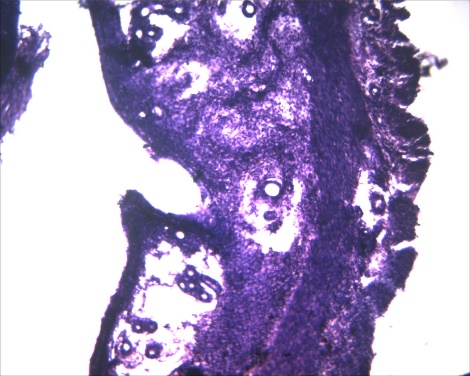


***Xylopia aethiopica* 3 Uterus:** No visible lesions seen. Glandular structures seen within the endometrium (arrows)

**Figure S1:** Histopathology of Ovaries and uterus of control and all treated groups
